# How artificial intelligence might disrupt diagnostics in hematology in the near future

**DOI:** 10.1038/s41388-021-01861-y

**Published:** 2021-06-08

**Authors:** Wencke Walter, Claudia Haferlach, Niroshan Nadarajah, Ines Schmidts, Constanze Kühn, Wolfgang Kern, Torsten Haferlach

**Affiliations:** MLL Munich Leukemia Laboratory, Munich, Germany

**Keywords:** Cancer, Haematological cancer

## Abstract

Artificial intelligence (AI) is about to make itself indispensable in the health care sector. Examples of successful applications or promising approaches range from the application of pattern recognition software to pre-process and analyze digital medical images, to deep learning algorithms for subtype or disease classification, and digital twin technology and in silico clinical trials. Moreover, machine-learning techniques are used to identify patterns and anomalies in electronic health records and to perform ad-hoc evaluations of gathered data from wearable health tracking devices for deep longitudinal phenotyping. In the last years, substantial progress has been made in automated image classification, reaching even superhuman level in some instances. Despite the increasing awareness of the importance of the genetic context, the diagnosis in hematology is still mainly based on the evaluation of the phenotype. Either by the analysis of microscopic images of cells in cytomorphology or by the analysis of cell populations in bidimensional plots obtained by flow cytometry. Here, AI algorithms not only spot details that might escape the human eye, but might also identify entirely new ways of interpreting these images. With the introduction of high-throughput next-generation sequencing in molecular genetics, the amount of available information is increasing exponentially, priming the field for the application of machine learning approaches. The goal of all the approaches is to allow personalized and informed interventions, to enhance treatment success, to improve the timeliness and accuracy of diagnoses, and to minimize technically induced misclassifications. The potential of AI-based applications is virtually endless but where do we stand in hematology and how far can we go?

## Introduction

Over the last 15 years, comprehensive diagnostics in leukemia and lymphoma has become increasingly challenging. In order to follow the guidelines of the World Health Organization (WHO) classification, the results from different fields, including cytomorphology, cytogenetics, immunophenotyping, and molecular genetics, have to be combined to establish a diagnosis. Gains in throughput from the introduction of next-generation sequencing (NGS) technologies and the accompanied broadening of the analytical spectrum in molecular genetics have boosted the value of molecular genetic results for diagnostics, as indicated by the revision of the WHO classification of leukemias and lymphomas in 2017 (Ref. [1]).

The plethora of available molecular information has broadened the landscape in leukemia and lymphoma diagnostics and has led to new insights in the underlying biology of the respective diseases, provoking a shift in diagnostics from phenotype to genotype. Moreover, the identification of an increasing list of diagnostic and prognostic markers, the refined estimate of inter-individual variability, and the ongoing effort to establish correlations between different layers of information that might eventually lead to improved targeted therapy options, are paving the way for personalized medicine. In parallel, it is indisputable that the data collection process goes digital, allowing the automated integration of different test results and easy access for all involved stakeholders. This journey also offers the opportunity to share information between clinical and genomic experts from multiple institutions, facilitating the assignment of patients to specific clinical trials or targeted treatment options [2]. Hence, the journey goes from analogous to digital and from phenotype to genotype.

Digital data is also a basic prerequisite for the application of emerging artificial intelligence (AI) techniques. Together with deep learning (DL) and machine learning (ML), AI is currently a buzzword across almost all scientific disciplines and has the potential to revolutionize diagnostic approaches in hematology. With the dramatic performance improvements in the last years, AI is at the brink to be introduced into routine diagnostics to enhance diagnostic methods but even more to facilitate disease classification and guidance of treatment. One exciting prospect is the development of digital twins to forecast cancer trajectories and to predict the potential impact of different therapeutic strategies in silico. The evaluation of these simulations might help to select the most promising interventions for each individual patient, minimizing side effects and the risk of complications [2, 3].

Here, we try to speculate what will happen in the next five years, how the landscape of leukemia diagnostics will be influenced by ML technologies, and how the future integration of AI-based methods will shape routine diagnostics in hematology. It is not our intention, to comprehensively review recent advances of the last five years but we like to highlight ML applications that are already being used, at least in research. Five years from now, it might be interesting to see where we were too optimistic and whether what we currently anticipate becomes a reality. We might even have AI-based methods so advanced, the complexity and capability of which, we are currently incapable of contemplating.

## Quick introduction to the principles of machine learning

**Fig. 1:**
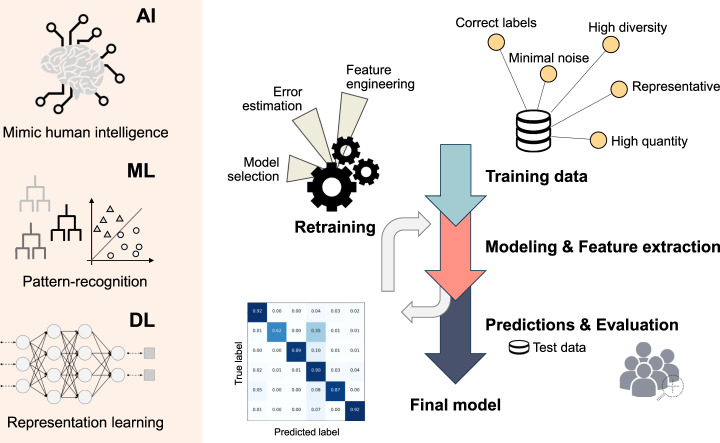
Overview of the different domains and the process of supervised learning. The left side represents the different domains of supervised learning going from artificial intelligence to machine learning and finally deep learning. The right side depicts the process of supervised learning. At the top right corner, the requirements for a good training data set are listed. The data is used for automated feature extraction, leading to the generation of a model, the performance of which is evaluated by its capability to correctly predict the labels of unseen instances (= test data). Based on the evaluation outcome, the model is retrained to refine the selected features and to optimize the model. After several rounds of retraining the final model emerges. AI artificial intelligence, DL deep learning, ML machine learning.

Due to the widespread interest and success of AI-based applications, the terms: artificial intelligence and machine learning, resound throughout the varying scientific disciplines, while often being used interchangeably in medicine. However, whereas AI strives to simulate human behavior and intelligence, ML, as a subdomain of AI, refers to the automatic detection of patterns and associations within the data (Fig. 1). DL, as a subfield of ML, allows layered neural networks to learn an abstract representation of often very complex data sets. AI and ML are not new and already in the early years the potential, risks and limits of AI have been hotly debated [4].

The remarkable improvement in technology in the biological field, especially for high-throughput methods, such as next-generation sequencing (NGS), has led to the faster generation of high quality data for a fraction of the original costs, resulting in the increased availability of digital data (=big data). Nonetheless, medical image classification, as an example for supervised learning, has benefitted the most from the introduction of ML methods to medicine so far [5, 6]. The advantage is the model’s adaptability and the fact that ML performs a task automatically on experience without getting explicit instructions, on a scale exceeding the capacity of the human brain. The algorithm is trained with a massive amount of data, requiring little human intervention, except for providing the correct class label to each image, and then left to extract relevant features and to draw its own connections, ultimately developing a set of rules and associations. The performance of the method is evaluated by its capability to predict the correct labels for a set of images not used in the training process (Fig. 1). The different types and techniques of ML in hematology have recently been reviewed [7].

The selection of an adequate training and test data set, in terms of quantity and quality, is crucial to obtain reliable results that are reproducible in real world scenarios and agnostic to the location and patient population (Fig. 1). In medicine, the training data sets are inevitably unbalanced because of the different prevalence of certain phenotypes and diseases in general but also in different subpopulations. The data sets on which the algorithms are trained, can be biased by a multitude of confounding factors that are not always obvious to the developers. Hence, it is essential to capture as much variability as possible by collecting a very diverse training set, reducing the risk of overfitting and increasing the likelihood of strong performances even for unseen instances. Ideally, the developed ML method should be transferable between hospitals and/or laboratories without loss of quality. However, due to the current lack in standardization for the operating procedures, minor adjustments are most likely unavoidable.

## Current applications and advances

### Cytomorphology

For more than 150 years, cytomorphology has been the backbone of hematological diagnostics, which is still true today. If an aberrant blood count is detected, cytomorphologic examinations are performed first, providing an initial diagnosis and guiding other diagnostic methods such as cytogenetics, immunophenotyping or molecular genetics to substantiate the result. However, the obtained preliminary diagnosis is solely based on the phenotype, and its correctness depends on pre-analytic procedures, as well as on the experience and capabilities of the personnel to accurately detect the aberrant cells, even very rare types. Hence, an automated pre-processing and evaluation of the digital microscopic images could benefit the reproducibility of results and would allow the hematologists and pathologists to focus on edge cases that do not fit the standard pattern, reducing the overall workload.

In the era of digital microscopic imaging and ML technologies, automated image processing, data analysis, and classification have become feasible. Initial attempts started with the segmentation of pre-processed images, followed by object identification, feature extraction, and lastly the classification of the different cell types. For the classification, different techniques have been applied ranging from support vector machines, to random forests, and artificial neural networks (ANN). With the increasing availability of digital images and the introduction of DL, the steps are less strictly separated, relying on the algorithm to differentiate between artefacts and informative material, as well as to extract relevant and meaningful features with limited human interference.

With respect to peripheral blood cells, the spectrum of successfully applied approaches ranges from the automated counting of white and red blood cells [8], to the differentiation between myeloblasts and lymphoblasts [9], and the simultaneous classification of different lymphoid cell types [10–12], as well as 17 (ref. [13]) and 21 (ref. [14]) cell types of different lineages and maturation states, including rare and malignant leukemia cells, to name a few. The automated identification and annotation of individual cells also forms the basis for the classification of different types of AML [15], the discrimination of reactive and MPN samples [16], and the differentiation of malignant and healthy cells for ALL diagnosis [17, 18] and ALL subtype classification [19, 20]. The quality of the results of each approach depends heavily on standardized pre-analytic, analytic and post-analytic parameters.

However, while in peripheral blood smears the cell density is sufficiently low to readily identify individual cells, the interpretation of bone marrow smears is much more difficult. Therefore, a pre-screening of the microscopic image is necessary to identify areas of high quality and single cell resolution. Here it is important that the areas are selected from different parts of the image to ensure the detection of all malignant cells, even if this means compromising on quality. Due to the increased complexity of the task, it is not surprising that even DL models for assisted interpretation of bone marrow smears [21, 22] could only yield moderate results so far (Table 1). Recently, digital pathology, which encompasses the digitization of histology glass slides, has emerged as a powerful tool for cancer diagnostics in general but also for diagnostics in hematology [23], largely benefitting from the introduction of DL for whole slide image analysis [24].

**Table 1:** Overview of the different diagnostic tests, the current challenges and known confunders for the clinical implementation of AI-based methods, and the requirements for a successful implementation.

	Cytomorphology	Cytogenetics	Immunophenotyping	Molecular genetics
Method	Microscopy	Chromosome banding analysis	Multiparameter flow cytometry	Genomic analysis
Aim	Identification and characterization of cell populations based on morphology	Identification of cytogenetic abnormalities	Identification and characterization of cell populations based on light-scattering properties and antigen expression patterns	Identification of individual molecular profiles
Challenges	Differentiation between artefacts and informative material (=cells)	Identification and selection of individual chromosomes	Accurate representation and transformation of the raw data	Data matrix usually sparse and informative signals might be lost in noise
	Correct identification of borders (very dense regions with overlapping cells)	Correct identification of structural abnormalities		Meaningful combination of various data types and differentiation between absence of information (e.g., insufficient coverage) and true negative results
	Extraction of features that allow the differentiation of maturation states of the same cell type			Lack of knowledge for annotation and interpretation of variants in coding and especially non-coding regions
Confunders	Resolution, image capturing, image cropping are not standardized between laboratories	Different banding and staining methods	Different methods for data pre-processing and data/image transformation	Plethora of methods for the identification of features (CNV, SV, SNV, Fusions, etc), data transformation, and dimensionality reduction with limited concordance and individual biases
	Unbalanced data sets for training with rare cell types being underrepresented	Unbalanced data sets for training with rare structural abnormalities being underrepresented		
Requirements for final implementation	Time and cost efficient digitization of glass slides		Harmonization and standardization of used antibodies	Harmonization of gene panels
	Standardized, automated systems for the recording of digital microscopic images			Standardization of analysis pipelines and variant interpretation
	Balanced training data capturing as much biological and technical variety as possible			

### Cytogenetics

Another diagnostic method in hematology that relies on the analysis and interpretation of morphological features is cytogenetics. Chromosome banding analysis has long been used in hematology and is the gold standard to identify cytogenetic abnormalities that allow the stratification of patients into disease subtypes with distinct prognosis. The patient-specific information is retrieved from the classification of chromosomes by size and banding as displayed in a karyogram. However, the generation of an accurate karyogram strongly depends on the quality of the captured metaphases and chromosomes, for which viable cells have to be grown and arrested in the metaphase stage of cell division. Subsequent banding and staining of the chromosomes is essential to highlight the details of diagnostic importance and to identify normal and abnormal chromosomes. However, karyotyping is a very time-consuming and complex task and a high degree of automation is desirable. For more than 30 years, various analysis systems for automated metaphase capturing and semi-automatic and/or interactive karyotyping have been available. These systems have been increasingly useful in classical human genetics, especially in prenatal diagnostics.

The challenges for automated karyotyping are multifarious and not yet fully resolved. First, individual chromosomes have to be identified and selected, excluding artefacts and overlapping or touching chromosomes from the downstream analysis. Already in 2007, an automated workflow was proposed [25] but most of the procedures still require some manual curation to avoid extensive discard and the artificial creation of abnormal chromosomes due to unfortunate cutting of overlapping chromosomes [26] (Table 1). It follows the labeling of the separated chromosomes and their assignment to the respective position in the karyogram. For an automated procedure, the identification of an optimal and small chromosome feature set is key for accurate performance and robustness. Common features considered for labeling include shape and size of the chromosome, the centromere location, and the unique banding pattern profile. Especially the banding pattern has been researched intensively to efficiently compute the profiles as a prerequisite for chromosome classification.

Over the last 20 years, various chromosome classifiers have been developed, ranging from ANN [27, 28], to competitive neural network teams [26], wavelet transform based linear discriminant analysis [29], and different versions of (deep) convoluted neural networks [30–34], achieving an accuracy from 85.2% to 98.6%. The classification often improved by correct alignment and orientation of the chromosomes along the vertical axis as a pre-processing step. Misclassification usually involved chromosomes, very similar in size, shape, and appearance and, hence, are challenging to differentiate, even for human professionals.

In tumor cytogenetics, chromosome anomalies, including numerical and structural abnormalities, are quite common and pose a further challenge for automated approaches. Numerical abnormalities usually involve normal chromosomes and, hence, most methods can be extended quite easily for this task. Structural abnormalities on the other hand are more challenging, due to the huge variety of possibilities and the sometimes limited available training material. However, promising early results show that also structurally abnormal karyograms might be detectable in an automated fashion in the future [34].

### Immunophenotyping

Besides cytomorphology, multiparameter flow cytometry (MFC, immunophenotyping) is the central method for the diagnosis of leukemias and lymphomas. MFC uses fluorescence dye-conjugated monoclonal antibodies, targeting diagnostically relevant antigens, to analyze cell populations based on their light-scattering properties and antigen expression patterns. Specific software automatically measures and captures the expression of the respective fluorescent dyes. Subsequently, human experts apply a sequential gating procedure to large sets of bidimensional plots to identify and label cell populations of interest. Although this method seems much less subjective than cytomorphology or histology, all the steps are error prone, influencing or even biasing the later interpretation. While standardized procedures are in place to control quality of sample preparation and measurement, the interpretation still relies on expert knowledge with inherited inter-observer variability. Thus, to reduce the dependency on expert knowledge and to increase reproducibility of data interpretation the implementation of automated procedures is desirable (Table 1).

An attempt in this field was conducted by Zhao et al. [35], who used self-organizing maps of light emission profiles as an input for a deep convolutional neural network to differentiate between healthy and neoplastic samples, as well as classification of mature B-neoplasm subtypes. Different clustering and ML techniques have been applied for joint cell clustering and identification of anomalous sample phenotypes for various hematologic malignancies [36], including AML samples [37, 38] and lymphomas [39–41]. Angeletti [42] applied a genetic algorithm to differentiate between AML and control samples and Bigorra et al. [43] could show that neural network approaches yield the highest accuracy for the differentiation between healthy controls, virus-infected samples and CLL patients. Only few approaches [44, 45] have attempted to use flow cytometry data for the classification without preceding image transformation. While Biehl et al. [44] used generalized matrix relevance learning vector quantification to separate AML patients from healthy controls, Müller et al. [45] applied a XGBoost model to assign lymphoma samples to their respective subtype.

Moreover, AI-based methods have been applied to accelerate, harmonize, and standardize interpretation of minimal residual disease (MRD) measurements obtained by flow cytometry, predicting the outcome of AML and MDS patients with high accuracy [46, 47].

### Molecular genetics

While the other fields integrate AI technologies to mimic human intelligence and to reproduce the knowledge of experienced diagnosticians, clinical molecular genetics aims to implement ML-based methods to perform tasks that are impractical for humans to do. With the introduction of high-throughput sequencing techniques and the accompanied analysis of large gene panels or even whole genomes and transcriptomes, molecular genetics has entered the realm of big data (Table 1). A single human genome contains 2x ~3.2 billion nucleotides worth of information and mining the data to obtain clinically relevant insights soon becomes cumbersome.

Basically every step in clinical genomic analysis could benefit from the integration of ML and DL methods [48, 49], including, but not limited to, the recognition of DNA sequence patterns [50, 51], variant calling [52], and variant effect prediction [53, 54] and classification [55]. Especially variant interpretation becomes more and more important with the increase in analyzed genes. Prioritization of causal and clinically actionable genetic variants is required for clinical decision-making and forms the basis for automated disease classification.

For some entities, the current WHO classification only mentions molecular genetic markers as a footnote so far but there is increasing recognition of the diagnostic relevance of the broader genetic context. Sequencing of a predefined set of gene regions associated with the suspected disease is currently favored, as opposed to whole exome sequencing, owing to decreased costs, to reduce turn-around times, and to limit an overburden of information that is not clinically actionable. The obtained mutational profile can be enriched by integration with phenotypic changes and clinical data as recently done by Nagata et al. who applied Bayesian ML techniques to identify diagnostically and prognostically relevant associations between genetic variants and cytomorphological changes in myelodysplastic syndromes (MDS). ML algorithms have also been applied to integrate mutational data, peripheral blood values, and clinical data into a geno-clinical model to differentiate various bone marrow disorders [56, 57]. However, the long-term goal might be to solely rely on molecular genetic data for disease and subgroup classification as previously done by different groups [58, 59].

Applying ML-based methods to molecular genetic data is not only relevant for clinical diagnosis but also for prognostication and the prediction of drug-responses. Wagner et al. [61] combined the molecular results of different databases in an ANN to identify a prognostic 3-gene signature that separated AML patients of European LeukemiaNet (ELN) strata in subgroups with different survival probabilities. A supervised ML approach applied to an *NPM1*^mut^ AML cohort identified clinically important mutations which were combined to a genetic score to pinpoint patients who are at high risk of relapse [62]. Supervised machine learning identified features that reliably assigned AML patients with *RUNX1-RUNX1T1* to favorable and poor risk classes [63]. In an MDS cohort an a priori market basket analysis algorithm was used to identify molecular signatures strongly associated with response to hypomethylating agents [64].

Although, the clinical application of transcriptome analysis is usually limited to the quantification of expression of a handful of genes by qPCR, different studies have also demonstrated the combined strength of larger gene panels and DL-based approaches for patient classification [60], biomarker detection [65], and predicting clinical response to anti-cancer drugs [66]. To overcome the difficulties presented by bulk gene expression data, DL has been applied to estimate cell type compositions from tissue expression profiles [67].

## Current challenges for the clinical implementation of AI-based methods

As outlined in the previous chapters, hematology could benefit substantially from the application of AI-based approaches to reduce the workload, to merge the knowledge of different experts, to decrease turn-around times, and to minimize technically induced misclassifications. The accurate interpretation of results depends a lot on a person’s level of experience and reliable AI-based clinical decision-support systems could install confidence in unexperienced clinicians, especially for morphology-based diagnostics. Different ML-based applications address these points, with the automated classification of cells and chromosomes from digital microscopic images nearly reaching expert clinical accuracy.

However, the performance of the algorithms largely depends on the availability of extensive standardized digital data to train the algorithms. The degree of workflow automation and hence, the homogeneity and reproducibility of the obtained data, varies between the different fields, potentially biasing the results (Table 1). Moreover, various parameters, such as the selected staining technique, the used antibodies or the selected genes and hotspots of a testing panel, often differ between different institutions, laboratories, and hospitals, impeding a generalization of the developed methods (Table 1). Hence, interlaboratory validation studies are necessary for a successful application and transferability of ML models and comprehensive guidelines have to be established to ensure standardized method outputs that can subsequently be fed into the ML models.

For some instances, the available training data is also limited in its complexity, capturing only part of the biological variability (Table 1). In cytomorphology, for example, it is challenging to obtain sufficient training material of rare cells, restricting the algorithm’s capability to extract meaningful features and to reliably identify the phenotype. The same applies to immunophenotyping and to rare chromosomal abnormalities in tumor cytogenetics. In molecular genetics, analyzed gene panels are often inconsistent among patients, depending on the suspected diagnosis and associated genes. Due to the sometimes-limited training material, there is a high risk of overfitting and the accidental fitting of confounders, resulting in significantly worse performances for unseen data. In addition, it is necessary to pay attention to avoid unintended discriminatory bias and failed generalization to new populations.

The benefit of AI technologies is the potential to improve the performance by constant learning, raising at the same time the unprecedented question about how to regulate such a machine. Initial guidelines have already been created around the globe [68]. However, one as yet unanswered question is the question of responsibility if an algorithms gets a diagnosis wrong. Most likely, there will not be an all-encompassing answer but each occurrence will have to be evaluated independently, taking into account the exact circumstances. Due to the initial poor results of AI-based approaches, many potential users are sceptical and successful, robust implementations, validated by clinical studies, will be necessary to eliminate the doubts. Furthermore, there is the ‘black box’ or ‘explainability’ problem of DL methods, allowing humans to comprehend the decisions of the algorithms only to a limited extent, undermining the scientific value of the method. The shortcomings of current ML-based methods in healthcare and potential solutions have been widely discussed [69–71]. Due to recent efforts, various methods exist to improve the transparency of an applied DL model [72], creating the neologism of ‘explainable artificial intelligence’ and paving the way for the clinical use.

## Opportunities and outlook

**Fig. 2:**
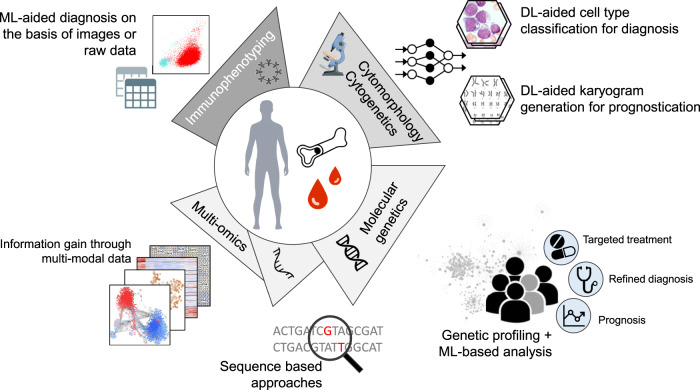
Overview of implemented and potential ML applications in hematology. The central part of the figure displays current and future diagnostic tests and methods, while the outer part illustrates the various data types and the potential clinical impact of ML-based applications and analyses. DL deep learning, ML machine learning.

As indicted before, the best opportunities for a fast implementation of ML-based technologies into routine diagnostic workflows offer cytomorphology and cytogenetics (Fig. 2). With the recent integration of DL methods, the accuracies of the different methods are close to expert level, offering the possibility of faster and more accurate sample processing, bringing expertise to the fingertips of less experienced hematologists. Although the current AI-based applications in flow cytometry are less extensive and have not reached clinically acceptable accuracies in all domains, an automated workflow could potentially lead to more standardized and reproducible results (Fig. 2).

In contrast to the other fields, the opportunities and possibilities in molecular genetics do not solely rely on the optimization of AI-based methods or an increase in standardized training data sets but also depend on the creation of comprehensive data collections and large-scale data mining efforts to improve the understanding of individual sequence variants and complex molecular constellations (Fig. 2). Comprehensive assays such as whole genome sequencing (WGS) and whole transcriptome sequencing (WTS) have great and so far mostly unexplored potential to revolutionize diagnostics of hematologic malignancies. Due to the high dimensionality and multi-modality, genomic data sets are the perfect candidate for DL explorations and can be used to refine diagnosis, further develop classification systems, identify prognostic factors, and to provide targets for individualized therapy (Fig. 2). Especially the identification of significant interactions and regulatory mechanisms are of tremendous interest. Dynamic mechanistic models can be used to analyze signaling regulations and even predict the effects of targeted therapies for personalized treatments [73]. Here, AI-based methods can also help to filter the clinically most relevant pathways [74]. Moreover, integrating molecular profiles and networks with deep, longitudinal physiological data and AI methods opens up the possibility for early detection of disease transition, prediction of clinical outcome, and the design of personalized treatment strategies [75, 76]. Another proposed solution to the challenge of efficiently linking an individual’s molecular profile to a compatible drug treatment is based on constructing Digital Twins [3]. The idea behind a digital twin in medicine is to build a computational model of a patient, which can be modified in silico, testing different treatments more quickly, economically, and safely than is possible in real life. The concept could be extended to in silico clinical trials, testing new drugs for a fraction of the current cost without putting patients at risk. The accuracy of such models could further be improved by adding multi-omics data (Fig. 2). Different ML-based methods have been developed for multi-omics analysis to conquer challenges such as data integration, biomarker discovery, and the identification of therapeutic compounds [77, 78]. The integration of multi-omics data opens up the opportunity for a more comprehensive diagnosis with a tailored treatment based on an individual’s genetic make-up.

## Conclusions

AI-based technologies continue to transform our everyday life and increasingly also different sectors of health care, including hematology. On one hand, the implementation of ML methods will aid clinicians in their analysis and interpretation of the data, increase objectivity and accuracy in the work-up, while on the other, the collected and the integrated knowledge will aid less experienced doctors in guiding their decision-making process. Here, regulated integration of new applications is important to ensure that patients are neither exposed to flawed interventions with potentially harmful effects nor denied access to beneficial innovations. Furthermore, in the future, molecular data, in combination with AI-based methods, might be superior to the phenotype-based standard methods in hematology, potentially defining new therapeutic approaches, yielding informed treatment decisions. Over the next five years, we will see an increasing need for AI approaches to integrate multi-modal patient data and treatment options as it becomes impossible for human intelligence to capture all this. However, in any field the prerequisite for the integration of ML-based methods is a reproducible and accredited data generation workflow. Only standardized outputs can be used for automated processing and provide results that, in combination with clinical judgment, can be used for diagnostics, for prognosis, and for therapy management in the context of precision medicine.

Despite the recent success of AI-based technologies in medicine, no system is infallible and results always have to be examined critically. It is not the intention to replace doctors with AI technologies but it would be negligent to ignore their potential. We need the support of AI to fulfill our primary goal: to provide patients with the best possible care. It is our duty to use all the means at our disposal to achieve this.
